# Hepatoprotective role of ascorbic acid against fenvalerate-induced histopathological, ultrastructural, and antioxidant disruptions in *Ctenopharyngodon idella*

**DOI:** 10.1016/j.toxrep.2025.101978

**Published:** 2025-02-28

**Authors:** Smriti Batoye, Sakshi Verma, Rajinder Jindal, Nidhi Srivastava

**Affiliations:** a Department of Zoology, Panjab University, Chandigarh 160014, India; b Department of Geography, Earth, and Environmental Science, University of Northern British Columbia, Prince George, BC V2N 4Z9, Canada; c Zoology Department, Hans Raj Mahila Maha Vidyalaya, Jalandhar 144008, India; d Department of Zoology, Maharaja Agrasen University, Baddi, Solan, HP 174103, India

**Keywords:** *pyrethroid*, *fenvalerate*, *ascorbic acid*, *antioxidant enzymes*, *transmission electron microscopy*, *histopathology*, *degree of tissue change*

## Abstract

*Ctenopharyngodon idella*, a herbivorous fish, is widely used in aquaculture to control aquatic weeds. Owing to its significant role, the present study investigates the protective effects of ascorbic acid (AA) against fenvalerate (FEN) toxicity in the liver of *Ctenopharyngodon idella.* Dietary AA supplementation (1000 mg/kg diet) was tested against 1.2 and 2 µg/L of FEN and fish were dissected on the 15th, 30th, and 60th day of the experiment. The results revealed a significant (p < 0.05) increase in liver antioxidant enzyme levels (superoxide dismutase, catalase, glutathione peroxidase, glutathione-S-transferase, and reduced glutathione) on the 15th and 30th days of FEN treatment followed by a decrease on the 60th day as compared to control group. While as, the malondialdehyde level was elevated throughout the experiment. Histopathological analysis revealed severe liver damage in FEN-treated fish, with notable infiltration of sinusoids, necrosis, and pycnotic nuclei, resulting in a mean degree of tissue change (DTC) value of 117.12 ± 1.27 at 2 µg/L of FEN on the 60th day of the experiment. Transmission electron microscopy displayed significant anomalies, including glycogen depletion, fragmented rough endoplasmic reticulum, swollen mitochondria, loss of heterochromatin, and necrotic hepatocytes with disrupted cytoplasm. However, dietary AA supplementation significantly minimized antioxidant enzyme activity and reduced liver pathology in FEN-treated fish, demonstrating its hepatoprotective efficacy. The study concludes that AA supplementation is recommended in aquaculture systems to mitigate the adverse effects of FEN.

## Highlights

• FEN induces hepatotoxicity in *C. idella*, causing oxidative stress and liver damage.
• Elevated SGOT, SGPT, and ALP levels indicate FEN-induced liver dysfunction.
• AA supplementation mitigates FEN toxicity, demonstrating hepatoprotective effects.
• AA’s antioxidant properties reduce oxidative stress and serum markers.
• Histopathological analysis confirms AA’s protective role against FEN-induced liver damage.

## Graphical Abstract

**Figure:**
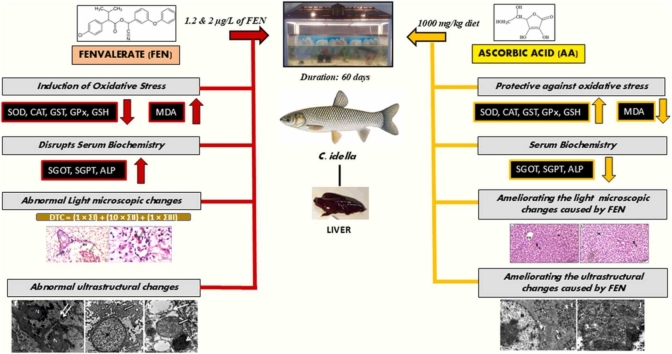

## 1 Introduction

Insecticides are extensively applied in agriculture to defend crops against pests that helps in enhancing food production and quality. However, their inevitable runoff into aquatic ecosystems poses a considerable threat to non-target organisms, mainly fish [1]. Among various insecticides, synthetic pyrethroids (SPs) have gained importance due to their high efficacy and relatively lower toxicity to mammals. However, their increased application has raised environmental concerns, as SPs can accumulate in aquatic environments, disrupting vital physiological processes such as metabolism, respiration, and reproduction in fish [2].

SPs are synthetic derivatives of pyrethrum, categorized into Type I and Type II based on the presence or absence of α-cyano group [3]. These insecticides now account for approximately 25 % of the global insecticide market and are widely used in agriculture, horticulture, and public health [4]. Despite their intended applications, the inadvertent release of SPs into aquatic environments through runoff and direct use in aquaculture (e.g., 2–5 µg/L for ectoparasite control) has heightened concerns about their toxicity to aquatic organisms [5]. The persistence of SPs in fish is exacerbated by their limited ability to metabolize these compounds due to the deficiency of hydrolyzing enzymes, leading to bioaccumulation and potential long-term effects on fish populations [6].

Fenvalerate (FEN), a Type II SP, is extensively used to control a broad spectrum of insect pests in agricultural and non-agricultural sites [7]. Despite its effectiveness, FEN is recognized as a highly toxic SP that poses severe risks to aquatic fauna, including fish, crustaceans, and zooplankton, as well as beneficial insects like honeybees [8]. Its mode of action involves disruption of voltage-gated sodium channels, ATPase-mediated transport systems, and γ-aminobutyric acid (GABA) receptors, leading to neurotoxicity and oxidative stress in exposed organisms [9].

Fish are widely used as bioindicators of aquatic environmental health, as they readily accumulate contaminants from water and their diet [10]. Given their nutritional importance as a source of high-quality protein, minerals, and vitamins, it is essential to ensure optimal water quality for sustainable aquaculture [11]. Biomarkers have become critical tools in ecotoxicological studies, allowing for early detection of contamination and assessment of pollutant impacts [12]. Among these, oxidative stress biomarkers have gained attention due to their ability to reflect on the cumulative effects of xenobiotics on aquatic organisms [13]. The response of antioxidant enzymes to toxicants varies depending on concentration, exposure duration, and species sensitivity [14]. The liver, a primary organ involved in metabolism, detoxification, and xenobiotic accumulation, serves a critical role for assessing insecticide toxicity [15]. Histopathological and biochemical analyses of liver tissue provide essential insights into the mechanisms of toxicity and potential protective strategies [16].

Several studies have investigated the toxic effects of FEN on development, hematological, biochemical, histopathological, and physical activities of different fish species, including *Channa punctatus* [17], *Clarias batrachus* [18], *Alburnus tarichi* [19], *Danio rario* [20], *Cirrhinus mrigala* [21], and *Oreochromis mossambicus* [22]. Zhang et al. [23] demonstrated that exposure to environmentally relevant levels of fenvalerate compromises immune function and decreases pathogen resistance in Chinese rare minnow (*Gobiocypris rarus*), emphasizing its ecological hazards. However, there is a lack of research focusing on *Ctenopharyngodon idella* (grass carp), a widely cultivated herbivorous fish species that plays a crucial role in aquaculture and aquatic vegetation management. Given its economic and ecological significance, understanding the impact of FEN toxicity in *C. idella* is essential for developing effective risk mitigation strategies. Additionally, although various antioxidants have been explored for mitigating pesticide-induced toxicity, many have shown limited efficacy or undesirable side effects [24], [25]. Ascorbic acid (AA), a water-soluble antioxidant, has demonstrated hepatoprotective and stress-mitigating properties [26], yet its potential in counteracting FEN toxicity in *C. idella* remains unexplored. Given that most fish species cannot synthesize AA endogenously and rely on dietary intake, optimizing AA supplementation to enhance stress tolerance and antioxidant defense in fish is of great significance [27]. AA plays a crucial role in mitigating pesticide-induced toxicity by neutralizing reactive oxygen species (ROS) and enhancing detoxification pathways [28]. Pesticides, including pyrethroids like fenvalerate, induce oxidative stress by generating free radicals, leading to lipid peroxidation, protein oxidation, and DNA damage [23]. AA counteracts these effects by scavenging free radicals, restoring antioxidant enzyme activity (e.g., superoxide dismutase, catalase, and glutathione peroxidase), and maintaining cellular redox homeostasis [11]. Additionally, AA supports hepatic detoxification by modulating phase I and phase II enzyme activities, thereby enhancing pesticide metabolism and excretion [25]. Studies have demonstrated that AA supplementation significantly reduces biochemical and histopathological alterations caused by pesticide exposure to fish, indicating its protective role in aquatic toxicology [29], [30], [31].

This study aims to address these research gaps by evaluating the toxic effects of FEN on the liver of *C. idella* and assessing the potential protective role of dietary AA supplementation. By assessing oxidative stress biomarkers, histopathological alterations, and biochemical parameters, this study seeks to provide new insights into the mechanisms of FEN toxicity and the effectiveness of antioxidant-based mitigation strategies in aquaculture.

## 2 Materials and methods

### 2.1 Fish

*C. idella* (14.21 ± 0.35 cm, 18.12 ± 0.89 g) were provided by ‘Sultan Fish Seed Farm’ in India and were acclimatized for 20 days in glass tanks (56 x 16 x 12″) with aerators and dechlorinated water. The experiments were carried out on fish according to the protocols included in the IAEC guidelines, Punjab University, Chandigarh (PU/IAEC/S/14/159). The laboratory-prepared basal feed, administered to fish twice daily at 2 % of their body weight, was formulated as outlined in Batoye et al. [32]. Briefly, during the acclimation phase, the fish received a basal diet made from groundnut oil cake (40 %), fish meal (25 %), rice bran (20 %), soybean meal (12 %), a vitamin and mineral blend (2 %), and starch (1 %), all mixed thoroughly. For the ascorbic acid (AA)-supplemented diet, 1000 mg of AA was incorporated per kg of the basal diet. The AA, sourced from Sisco Research Laboratories Pvt. Ltd., was a white powder with a molecular formula of C₆H₈O₆, a molecular weight of 176.13 g/mol, and a melting point between 190–192°C. The proximate nutrient composition of the fish diet, expressed as a percentage of dry weight (mean ± SE), included 90.26 ± 0.17 % dry matter, 37.04 ± 0.05 % crude protein, 10.26 ± 0.16 % ash content, and 7.36 ± 0.09 % lipids. During the acclimatization phase, one-third of the water was replaced twice weekly to eliminate waste and uneaten feed.

### 2.2 Experimental design

The commercial formulation of fenvalerate (20 % EC) manufactured by Shivalik Insecticides Limited, India was used. The 96-hour LC_50_ of FEN for *C. idella* was calculated using Probit analysis and found to be 5.9 μg/L [32]. For the chronic toxicity experiment, two sub-lethal concentrations of FEN - 1.2 µg/L (one-fifth of 96-hour LC_50_) and 2 µg/L (one-third of 96-hour LC_50_) along with a dose of ascorbic acid (1000 mg/kg bw/day) were chosen based on our previous research [32]. A total of 180 fish (10/replicate) were distributed into 6-groups (each group was maintained in 3 experimental replicates):

**Group-I:** control, without AA and FEN;
**Group-II:** orally administered with AA (1000 mg/kg) in the diet, no FEN;
**Group-III:** treated with FEN (1.2 µg/L);
**Group-IV:** treated with FEN (1.2 µg/L) + 1000 mg/kg diet AA;
**Group-V:** treated with FEN (2 µg/L);
**Group-VI:** treated with FEN (2 µg/L) + 1000 mg/kg diet AA.

The test was over 60 days in syntax plastic tanks (24″ x 12″ x 12″) with 100 L of water. Two fish from each experimental replicate (n = 6/group) were sacrificed by cervical dislocation on the 15th, 30th and 60th day of the experiment. Throughout the study, the water quality parameters were maintained at 24 ± 2 °C temperature, dissolved oxygen of 9.0 ± 0.50 mg/L, hardness of 90.34 ± 5.0 mg/L, and pH of 7.1–7.3. For the chronic toxicity test, water was replaced daily to maintain consistent fenvalerate concentrations in water throughout the exposure period.

### 2.3 Serum biochemistry

Blood samples were taken from the fish (n = 6 per group) and allowed to clot for 15–20 minutes at room temperature. Following coagulation, samples were centrifuged at 5000 rpm for 10 minutes to separate the serum, which was stored at −20°C until further analysis. Serum levels of glutamic oxaloacetate transaminase (SGOT), glutamate pyruvate transaminase (SGPT), and alkaline phosphatase (ALP) were measured using Reckon Diagnostics Pvt. Ltd. diagnostic kits (SGPT: CC2-ALT.17 N, SGOT: CC2-AST.16 N, ALP: CC2-ALK.002), following the manufacturer’s instructions. Absorbance readings for these parameters were taken using a Jenway 6305 UV/Vis spectrophotometer. SGOT activity (IU/L) was determined by mixing 1 ml of working solution with 0.1 ml of serum, followed by incubation at 37°C for 60 seconds. The reaction mixture, containing L-aspartate, α-ketoglutarate, malate dehydrogenase, and NADH, was measured for the decrease in absorbance at 340 nm at 30-second intervals for 2 minutes. For SGPT (IU/L), 1 ml of working solution was added to 0.1 ml of serum, and the reaction, involving L-alanine, α-ketoglutarate, lactate dehydrogenase, and NADH, was incubated at 37°C for 60 seconds. Absorbance changes at 340 nm were measured at 30-second intervals for 2 minutes. ALP activity (IU/L) was measured by adding 1 ml of buffered substrate to 0.02 ml of serum. The reaction mixture, containing para-nitrophenyl phosphate, was incubated at 37°C, and the increase in absorbance at 405 nm was measured every 30 seconds over 2 minutes.

### 2.4 Oxidative stress analysis

For oxidative stress analysis, fish (n = 6/group) from all the experimental groups were randomly selected and dissected out to remove the liver. The liver was washed in saline (0.9 % NaCl), homogenized in Tris-HCl buffer (0.1 M, pH 7.4, 4 °C) to obtain 10 % (w/v) homogenate. For post mitochondrial supernatant (PMS), again centrifuged (for 30 min at 1000 rpm, 4 °C) and stored at −30 °C.

SOD activity was quantified following Kono's [33] procedure. The assay mixture was prepared by combining 1.2 ml of 50 mM sodium carbonate buffer (pH 10.8) containing 0.1 mM EDTA, 0.5 ml of 96 µM nitro blue tetrazolium (NBT), and 0.1 ml of 0.6 % Triton X-100. The reaction mixture was incubated at 37 °C for 10 minutes, and the reaction was initiated by adding 0.1 ml of 20 mM hydroxylamine hydrochloride. The rate of NBT reduction, resulting from superoxide radicals generated via photoactivation of hydroxylamine, was monitored at 560 nm for 3 minutes. After the addition of 0.1 ml of the sample supernatant, the extent of inhibition of NBT reduction, caused by SOD, was recorded as the change in absorbance at 560 nm for 3 minutes. SOD activity was expressed as units per mg of protein.

CAT activity was assessed by measuring the decomposition of hydrogen peroxide (H_2_O_2_) using the method of Luck [34]. The reaction mixture consisted of 2.9 ml of H_2_O_2_-phosphate buffer and 0.1 ml of supernatant. The reduction in absorbance at 240 nm was measured at 30-second intervals over a 3-minute period. The rate of H_2_O_2_ decomposition was quantified as micromoles of H_2_O_2_ decomposed per minute per mg of protein.

GPx activity was determined spectrophotometrically at 340 nm, following the procedure of Mohandas et al. [35]. The reaction mix included 0.1 ml of 1 mM EDTA, 0.1 ml of 1 mM sodium azide, 1.49 ml of 0.1 M phosphate buffer (pH 7.4), 0.05 ml of 1 IU/ml glutathione reductase, 0.05 ml of 1 mM reduced glutathione, 0.1 ml of 0.2 mM NADPH, 0.01 ml of 0.25 mM H_2_O_2_, and 0.1 ml of the supernatant. The consumption of NADPH at 340 nm, reflecting the oxidation of NADPH to NADP, was recorded. GPx activity was expressed as micromoles of NADPH oxidized per minute per mg of protein.

GST activity was determined by monitoring the formation of CDNB-GSH conjugates at 340 nm, as described by Habig et al. [36]. The assay mixture contained 0.1 ml of 30 mM GSH, 0.1 ml of 30 mM CDNB, 2.7 ml of potassium phosphate buffer (pH 6.5), and 0.1 ml of supernatant. The GST activity was calculated based on the change in absorbance at 340 nm, and the results were expressed as micromoles of conjugate formed per minute per mg of protein.

The level of GSH was assessed using the method of Beutler et al. [37], with spectrophotometric measurement at 412 nm. GSH reacts with 5,5′-dithiobis-2-nitrobenzoic acid (DTNB) to form a yellow-colored complex, the intensity of which was used to quantify GSH levels. The GSH content was expressed as micromoles of conjugate formed per gram of protein.

MDA levels, indicative of lipid peroxidation, were determined following the procedure of Buege and Aust [38]. A volume of 0.25 ml of supernatant was incubated with 0.25 ml each of 150 mM Tris-HCl buffer (pH 7.4), 1.5 mM ascorbic acid, and 1.0 mM ferrous sulfate at 37 °C for 15 minutes. Following the reaction, 1.0 ml of 10 % trichloroacetic acid (TCA) and 2.0 ml of 0.375 % thiobarbituric acid (TBA) were added, and the mixture was heated in a boiling water bath for 15 minutes. After centrifugation at 3000 rpm for 10 minutes, the absorbance of the supernatant was measured at 532 nm. MDA levels were expressed as micromoles per mg of protein.

The protein content was quantified using the Lowry et al. [39] method, with bovine serum albumin as the standard.

### 2.5 Histopathological analysis

For histopathological examination, tissue processing followed the method described by Cengiz and Unlu [40]. The excised liver tissue was rinsed in saline solution and fixed using Bouin’s fixative. After fixation, the tissue was dehydrated through a graded alcohol series, cleared with xylene, and embedded in paraffin. Thin sections of 5 µm were then prepared, stained with hematoxylin and eosin, and viewed under a light microscope. Photographs were taken at the Central Instrumentation Laboratory (CIL) at Panjab University, Chandigarh. The degree of tissue change (DTC) was used to evaluate pathological lesions in the liver across all experimental groups. Three histological sections per fish were analyzed, and the DTC was calculated using the formula [41]:

DTC=(1×∑I)+(10×∑II)+(100×∑III)

where I, II, and III represent the total number of lesions at stage I (minor changes with no impact on tissue function), stage II (extreme and tissue function-impairing changes), and stage III (severe, irreversible damage). The mean DTC values (n = 18) were used to assess liver injury severity based on the following scale: normal tissue function (0−10), slight damage (11−20), moderate damage (21−50), severe damage (51−100), and irreversible damage (>100).

### 2.6 Ultrastructural analysis

For transmission electron microscopy (TEM), tissue samples were initially fixed in Karnovsky's fixative for 10–12 hours (pH 7.2–7.3, 4°C) and then rinsed in phosphate buffer. They were post-fixed in 1 % osmium tetroxide in 0.2 M phosphate buffer (for 1 hour at 4°C), followed by washing in 0.1 M phosphate buffer. The samples were dehydrated in a graded acetone series, embedded in Epon 812 resin, and polymerized. Ultrathin sections (50–60 nm) were obtained from the polymerized blocks using an ultramicrotome, mounted on copper grids, stained with uranyl acetate and lead citrate [42], and then examined under a TEM at AIIMS, New Delhi, India.

### 2.7 Statistical analysis

Data were analyzed using SPSS 18.0 software. The Kolmogorov-Smirnov test was employed to check the normal distribution of data obtained from oxidative stress and histopathological (DTC) assessments. Data with a normal distribution were analyzed using one-way ANOVA (Tukey’s post hoc test) and are presented as mean ± standard error. Statistical significance between groups was considered at p < 0.05.

## 3 Results

### 3.1 Serum biochemistry

**Fig. 1:**
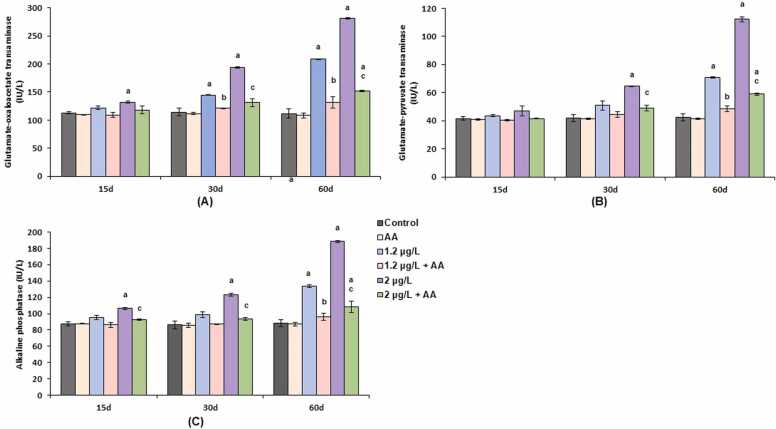
(A-C). Serum levels of GOT, GPT and ALP in *C. idella* exposed to FEN and orally administered with AA at different time intervals. Data are presented as mean±SE (six individuals). p < 0.05 is statistically significant, determined by one way ANOVA followed by Tukey’s post hoc test. ‘a’ indicates a statistically significant difference compared to the control. ‘b’: significant difference between FEN (1.2 µg/L) and FEN (1.2 µg/L) + AA. ‘c’: significant difference between FEN (2 µg/L) and FEN (2 µg/L) + AA.

Fish exposed to FEN alone showed significant increases in GOT, GPT, and ALP activities (Fig. 1A-C), which were influenced by both insecticide concentration and exposure duration. In contrast, control and AA-only groups displayed no significant changes. After 15 days, fish treated with a lower FEN concentration showed a minor, non-significant increase in GOT, while a higher concentration led to a significant (p < 0.05) 17 % rise. GPT levels exhibited a slight, non-significant increase at both FEN concentrations, and ALP levels rose non-significantly at the lower concentration, but significantly (p < 0.05) by 22 % at the higher dose. Co-administration with AA significantly (p < 0.05) reduced GOT, GPT, and ALP levels close to control values.

After 30 days, FEN exposure at 1.2 and 2 µg/L resulted in significant (p < 0.05) increases in GOT by 27 % and 70 %, respectively, while GPT levels increased by 54 % at the higher dose (Fig. 1A-B). ALP also rose significantly (p < 0.05) by 43 % at the higher concentration (Fig. 1A-B). AA supplementation effectively normalized GOT, GPT, and ALP levels in comparison to the FEN-only group. After 60 days, the highest increases in GOT and GPT activities were observed, with GOT rising by 87 % and 152 %, and GPT by 67 % and 165 % for the lower and higher FEN concentrations, respectively. ALP levels showed maximum increases of 114 % (188.29 ± 1.3) at the higher dose and 52 % (133.74 ± 1.95) at the lower dose, relative to control (87.99 ± 4.29). AA co-treatment significantly (p < 0.05) lowered GOT by 36.89 % and 46.03 %, GPT by 31.73 % and 47.55 %, and brought ALP levels close to control, restoring enzyme activities to near-normal levels (Fig. 1A-C).

### 3.2 Oxidative stress analysis

**Fig. 2:**
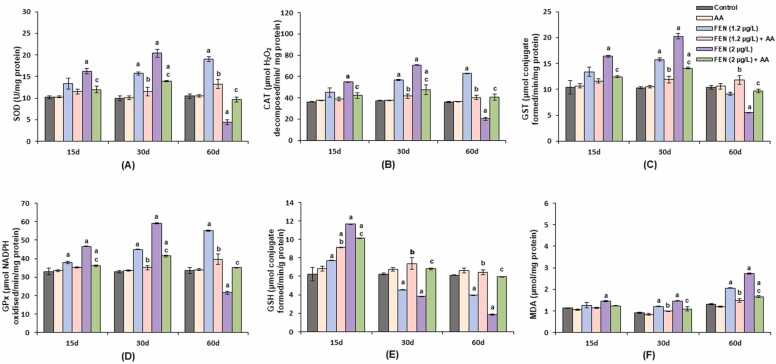
**(**A-F). Activity of SOD, CAT, GST, GPx, and levels of GSH and MDA, in the liver of *C. idella* exposed to FEN and orally administered with AA at different time intervals (n = 6). Data are presented as mean±SE (six individuals). p < 0.05 is statistically significant, determined by one way ANOVA followed by Tukey’s post hoc test. Tukey’s post hoc test. ‘a’ indicates a statistically significant difference compared to the control. ‘b’: significant difference between FEN (1.2 µg/L) and FEN (1.2 µg/L) + AA. ‘c’: significant difference between FEN (2 µg/L) and FEN (2 µg/L) + AA.

Fig. 2 (A-D) displayed non-significant change in the SOD, CAT, GPx and GST activity in the liver of *C. idella* orally administered with AA alone as compared with control. Similarly, GSH and MDA level also displayed a non-significant increase in the control and AA alone treated group Fig. 2(E and F).

#### 3.2.1 SOD

Exposure to 1.2 µg/L FEN demonstrated an increasing trend in SOD activity, which was found to be time and concentration dependent as shown in Fig. 2(A). SOD activity in the liver of fish treated with 1.2 µg/L FEN showed a non-significant increase on the 15th day while significantly increased (p < 0.05) by 57.72 & 82.1 % on the 30th and 60th day as compared to control. Whereas AA supplementation with 1.2 µg/L FEN significantly normalised the SOD activity in the liver as indicated by 13.74, 26.59 & 30.32 % decrease on 15th, 30th and 60th day respectively as compared to FEN alone treated group. In Figs. 2(A), 2 µg/L FEN significantly (p < 0.05) increased the SOD activity upto the 30th day by 104.96 % afterward on 60th day, it was declined by 58 % as compared to control. AA + 2 µg/L FEN decreased the activity of SOD by 26.29 & 31.68 % on 15th and 30th day respectively, whereas increased by 119.59 % on 60th day as compared to FEN alone treated groups.

#### 3.2.2 CAT

In Fig. 2(B), significant (p < 0.05) rise in the CAT activity was monitored on 30th and 60th day by 52.03 & 73.98 % respectively as compared with control on exposure to 1.2 µg/L FEN. 2 µg/L FEN exposure showed significant augmentation in CAT activity by 51 % & 88.82 % on 15th and 30th day respectively, over control values. Furthermore, it was found to be decreased below the control level by 43.98 %. Administration of AA with FEN exposure significantly (p < 0.05) decreased CAT activity near to control on the 15th day. On the 30th day, the CAT activity in the liver of AA + FEN (1.2 µg/L) and AA + FEN (2 µg/L) treated fish was decreased by 27.10 and 32.86 % respectively when compared with their respective toxicant alone treated groups. On the contrary, treatment with 1.2 µg/L FEN + AA in Fig. 2(B) for 60 days showed a significant (p < 0.05) decrease by 36.06 % whereas a significant increase was noticed by 101.03 % in fish treated with 2 µg/L FEN + AA as compared to the respective toxicant alone treated groups.

#### 3.2.3 GST

On the 15th day of the experiment, as shown in Fig. 2(C), exposure to 1.2 µg/L FEN caused a non-significant increase in GST activity. By day 30, GST activity had significantly (p < 0.05) increased by 53 %, while prolonged exposure (60 days) resulted in a non-significant decrease of 12 % compared to control. In the AA + FEN (1.2 µg/L) group, GST activity was reduced by 13.21 % and 24.39 % on days 15 and 30, respectively, compared to the FEN-only group. On day 60, AA co-treatment significantly (p < 0.05) increased GST activity by 29.63 % relative to the FEN-only group. Exposure to 2 µg/L FEN, as depicted in Fig. 2(C), significantly (p < 0.05) increased GST activity by 58 % on day 15 and 96.80 % on day 30. However, by day 60, GST activity had decreased by 47 % compared to control. With AA supplementation alongside the higher FEN concentration, GST activity decreased significantly by 24.08 % on day 15, approaching control levels. After 30 days, AA co-treatment led to a significant 30.41 % reduction in GST activity compared to the FEN-only group, though levels remained above control. By day 60, GST activity was significantly elevated by 76.50 % in the AA + FEN group compared to the FEN-only group.

#### 3.2.4 GPx

As shown in Fig. 2(D), exposure to 1.2 µg/L FEN led to a time-dependent increase (p < 0.05) in GPx activity, with elevations of 14.90 %, 37 %, and 64 % on days 15, 30, and 60, respectively, compared to control. The group receiving AA + 1.2 µg/L FEN exhibited significant normalization of GPx activity, with reductions of 7.40 %, 21.87 %, and 28.05 % relative to the FEN-only group. Exposure to 2 µg/L FEN significantly (p < 0.05) increased GPx activity by 41 % on day 15 and 80 % on day 30, followed by a significant (p < 0.05) 36 % decrease on day 60 compared to control. AA supplementation with higher FEN concentration facilitated significant recovery in GPx activity, which was near control levels by day 30. However, on day 60, GPx activity significantly increased by 63.28 % compared to the FEN-alone group.

#### 3.2.5 GSH

Exposure to 1.2 and 2 µg/L FEN for 15 days presented in Fig. 2(E), caused a significant increase (p < 0.05) in GSH content as compared to control. The maximum increase in the antioxidant level was noticed by 87 % when exposed to the 2 µg/L FEN. Thereafter, a declining trend in GSH level was encountered in both the concentrations of the toxicant on the 30th day. In Fig. 2(E), the liver showed maximum depletion in GSH content by 70 % on the 60th day in the group exposed to the FEN higher concentration, relative to control. On the 15th day, supplementation of AA + 1.2 µg/L FEN, significantly increased GSH level by 18.52 %, whereas fish co-treated with AA + 2 µg/L FEN showed a significant decrease by 12.87 % when compared to the respective FEN alone treated groups. After 30 days of treatment with AA + FEN (1.2 µg/L), an increase in GSH content was registered by 62.69 %, and treatment with AA + FEN (2 µg/L) by 78.74 % are shown in Fig. 2(E). After 60 days, AA supplementation significantly brought GSH level near to control as compared to FEN-only groups (Fig. 2E).

#### 3.2.6 MDA

Exposure to the 1.2 µg/L FEN for 15 days, caused a non-significant augmentation in the MDA level and a significant increase (p < 0.05) by 34 % on the 30th day as compared to their corresponding control in Fig. 2(F). A further significant (p < 0.05) increase of 59 % in MDA levels was observed on day 60. In contrast, as shown in Fig. 2(F), the MDA level in the AA + 1.2 µg/L FEN group was close to control on days 15 and 30, and by day 60, there was a significant decrease of 28.15 % compared to the FEN-only group. Exposure to 2 µg/L FEN significantly (p < 0.05) raised MDA levels by 29 %, 61 %, and 110 % on days 15, 30, and 60, respectively, compared to control. With AA co-treatment at 2 µg/L FEN, MDA levels were near control on day 15, and on day 30, they showed a significant reduction of 25.34 % compared to the FEN-only group. On day 60, fish treated with AA + 2 µg/L FEN exhibited a significant difference in MDA levels compared to both the FEN-only group and control.

### 3.3 Histopathological analysis

**Fig. 3:**
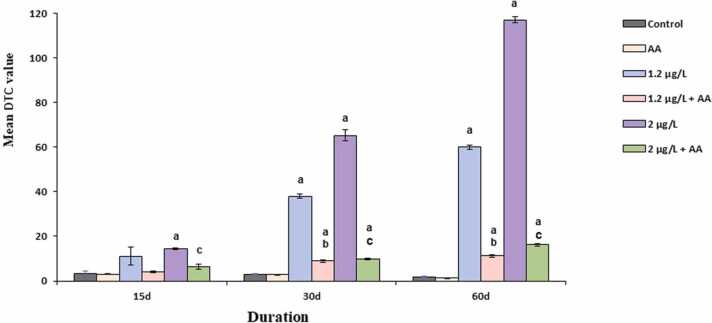
Degree of tissue change (DTC) calculated for liver of *C. idella* exposed to FEN and orally administered with AA at different time intervals. Data are presented as mean±SE. p < 0.05 is considered to be statistically significant, determined by one way ANOVA followed by Tukey’s post hoc test. ‘a’ indicates a statistically significant difference compared to the control. ‘b’: significant difference between FEN (1.2 µg/L) and FEN (1.2 µg/L) + AA. ‘c’: significant difference between FEN (2 µg/L) and FEN (2 µg/L) + AA.

**Fig. 4:**
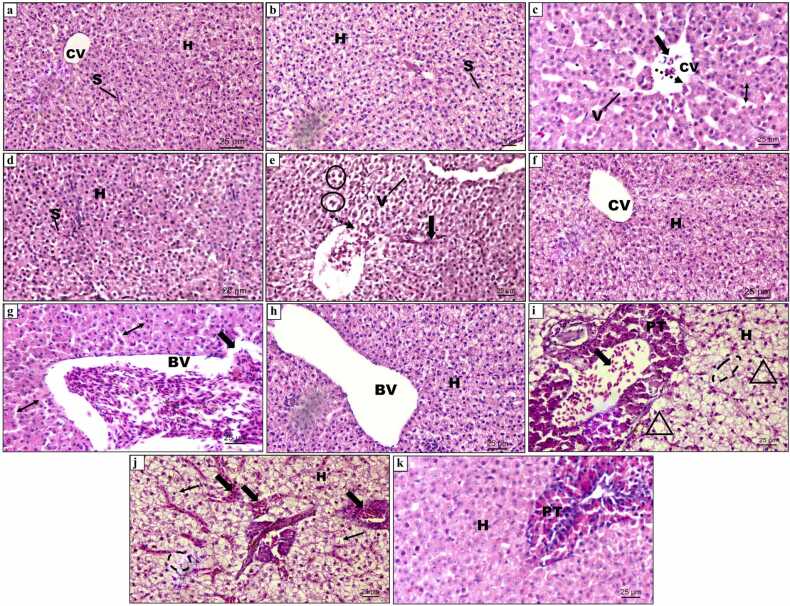
Haematoxylin and eosin stained sections of liver tissue of *C. idella* (a-b) control and AA alone treated groups respectively, showing polygonal hepatocytes with centrally located nucleus surrounding the well-defined CV and sinusoids inter-spread between hepatic cords; (c-f) exposed for 15 days: (c) 1.2 µg/L FEN exposure showing mild disruption in the endothelium of CV (dotted arrow), granular cytoplasm and vacuolization, lymphocytic infiltration (black block arrow), dilation of sinusoidal spaces (line arrow: double) and; (d) treated with AA + 1.2 µg/L FEN. Note the normal structure of hepatocytes. (e) 2 µg/L FEN exposure showing ruptured endothelium of CV (dotted arrow), steatosis (oval), infiltration in sinusoid (arrow), vacuolization; (f) treated with AA + 2 µg/L FEN depicting normalization of CV and hepatocytes; (g-k) exposed for 30 days: (g) 1.2 µg/L FEN exposure showing dilated hepatic sinusoidal spaces (line arrow: double), ruptured endothelium of blood vessel with infiltration (block arrow); (h) treated with AA + 1.2 µg/L FEN. Note the normal structure of hepatocytes and blood vessel; (i, j) 2 µg/L FEN exposure showing damaged pancreatic tissue with infiltration of lymphocytes (block arrow), hydropic degeneration (triangle), karyolysis (dotted oval), infiltration of lymphocytes in sinusoidal spaces (block arrow); (k) treated with AA + 2 µg/L FEN. Note the normal structure of pancreatic tissue and hepatocytes; blood vessel (BV), hepatocyte (H), central vein (CV), sinusoid (S), vacuolization (V), pancreatic tissue (PT).

The liver parenchyma in the control fish is composed of polyhedral hepatocytes comprising homogenous cytoplasm and centrally positioned nucleus, organised in cords around the central vein, arbitrarily distributed within the liver (Fig. 4a). The capillary sinusoids are interspersed between the hepatic cords. Fig. 4(a and b) showed no significant changes in control and AA alone treated fish liver and mean DTC values as presented in Fig. 3, Table 1 were found to be in the normal range (1.88 ± 0.26–3.26 ± 1.33) reflecting the normal functioning of the liver. The histological investigation discovered that FEN exposure displayed pathological lesions in the liver, such as vacuole formation, disruption of central vein, the appearance of nuclear polymorphism, sinusoidal dilation, lymphatic infiltration and hepatic degeneration. A total of 10 types of lesions including 5 (Stage I), 4 (Stage II) and 1 (Stage III) were recognized according to Camargo and Martinez [43] and are presented below:

**Table:** 

**Alterations**	**Stage**

Oedema	I
Sinusoidal dilation	I
Intracellular vacuolisation	I
Macrophage aggregates	I
Nuclear polymorphism	I
Lymphatic infiltration in blood vessels	II
Hydropic degeneration	II
Cellular rupture	II
Pycnotic nucleus	II
Hepatic necrosis	III

**Table 1:** Degree of tissue change (DTC) for the liver of *C. idella* treated with FEN and AA alone, and in combination at different time intervals.

Tissue	Duration (Days)	Control	AA	FEN (1.2 µg/L)	FEN (1.2 µg/L) + AA	FEN (2 µg/L)	FEN (2 µg/L) +AA
**Liver**	15	3.26 ± 1.33	3.09 ± 0.18	11.05 ± 1.06	4.13 ± 0.27	14.33 ± 0.43^a^	6.29 ± 1.09^c^
	30	3.05 ± 0.19	2.81 ± 0.15	38.02 ± 1.01^a^	8.90 ± 0.60^ab^	65.26 ± 1.46^a^	9.80 ± 0.20^ac^
	60	1.88 ± 0.26	1.27 ± 0.09	60.04 ± 1.02^a^	11.22 ± 0.66^ab^	117.12 ± 1.27^a^	16.16 ± 0.66^ac^

Where, AA: Ascorbic acid (1000 mg/kg diet)

‘a’ indicates (p < 0.05) difference with respect to control.

‘b’: FEN (1.2 µg/L) vs FEN (1.2 µg/L) + AA.

‘c’: FEN (2 µg/L) vs FEN (2 µg/L) + AA.

Fig. 4(c) showed mild disruption in the endothelium of central vein, granular cytoplasm, vacuolization, lymphocytic infiltration and dilation of sinusoidal spaces on exposure to 1.2 µg/L FEN for 15 days. Adding to these in Figs. 4(e), 2 µg/L exposure displayed steatosis, ruptured endothelium of central vein, infiltration in sinusoids, and vacuolization in hepatocytes. Moreover, the semiqualitative analysis displayed in Fig. 3, Table 1 revealed a significant interconnection between increasing concentration and damage to the liver as evidenced by 4.84 and 6.78-fold increase in mean DTC value in fish exposed to the lower and higher concentration of insecticide respectively, when compared to that of control. Conversely, concomitant treatment of AA with FEN in Fig. 4(d and f) significantly recovered these alterations, as evident from well-organised hepatocytes in cords with the normal structure of the central vein. Additionally, as mentioned in Fig. 3, Table 1 significant decrease in the values of DTC was observed in both the groups, and the values i.e., 4.13 ± 0.27 and 6.29 ± 1.09 for FEN (1.2 µg/L) + AA and FEN (2 µg/L) + AA respectively, were found to be near control (3.26 ± 1.33), thus signifying the usual working of the organ.

These FEN-induced histological changes in the liver were found to be more frequent, including many abnormalities from stage II, with the increase in the exposure period. Lower FEN concentration after 30 days of exposure as shown in Fig. 4(g), caused various damage, such as dilated hepatic sinusoidal spaces and ruptured endothelium of blood vessels with infiltration. The histopathological semiquantitative analysis in Fig. 3, Table 1 showed moderately damaged tissue as evinced by a DTC value of 38.02 ± 1.01 which is 12.46-fold higher than that of control. However, oral administration of AA with lower concentration of FEN reverts these alterations to approximately normal structure as that of control, as evident from well-organised hepatocytes around normal sinusoids and blood vessels displayed in Fig. 4(h). Also, the DTC value mentioned in Fig. 3, Table 1 was 8.90 ± 0.60, which was near control (3.05 ± 0.19). Fish exposed to 2 µg/L FEN after 30 days showed marked deteriorated changes in the liver and frequently involved stage II abnormalities. The histopathological anomalies encountered in this group include damaged pancreatic tissue with infiltration of lymphocytes, infiltrated sinusoidal spaces, hydropic degeneration and karyolysis (Fig. 4i and j). In Fig. 3, Table 1, the DTC value (65.26 ± 1.46) also depicted a significant (p < 0.05) increase in the damage to the tissue by 21.39-fold in comparison to control, thus reflecting the severe damage to the organ. On the other hand, AA along with FEN (2 µg/L) in Fig. 4(k) inferred marked recovery in hepatic lesions induced by FEN alone treated group. The DTC value (9.80 ± 0.20) was also found to be 6.65-fold lesser than that of the FEN alone group (Fig. 3, Table 1).

**Fig. 5:**
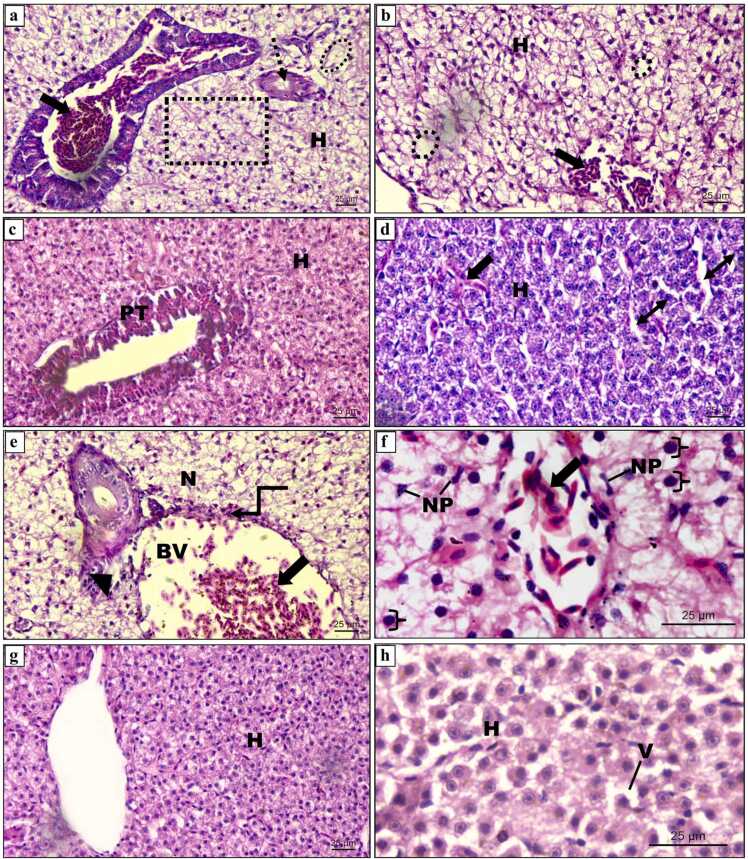
Haematoxylin and eosin stained sections of liver of *C. idella* exposed to FEN and orally administered with AA (60 days): (a-b) 1.2 µg/L FEN exposure showing damaged blood vessel (dotted arrow), hypertrophy of hepatocytes (rectangle), massive infiltration (black block arrow), karyolysis (dotted oval); (c-d) treatment with AA + 1.2 µg/L FEN. Note the normal organization of liver tissue with mild dilation (line arrow: double) and lymphatic infiltration in the sinusoids (black block arrow). (e-f) 2 µg/L FEN exposure showing necrosis, perivascular lymphatic cuffing (elbow arrow), heavy lymphatic infiltration in the blood vessel (black block arrow), perivascular oedema (arrow head), nuclear polymorphism (arrow), pycnotic nuclei (right brace); (g-h) treatment with AA + 1.2 µg/L FEN recovered the hepatocytes morphology except for vacuolization; hepatocyte (H), pancreatic tissue (PT), nuclear polymorphism (NP), blood vessel (BV), necrosis (N), vacuolization (V).

Marked lesions in the liver were observed when fish was exposed to FEN for 60 days. Exposure to 1.2 µg/L of FEN (60 days) in Fig. 5(a and b) showed destruction of the endothelium of blood vessel, hypertrophy of hepatocytes, karyolysis and massive infiltration. Massive infiltration and karyolysis were among the frequently encountered lesions and the DTC value (60.04 ± 1.02) showed severe damage to the tissue which was 31.93-fold higher than that of control (Fig. 3, Table 1). However, in AA and FEN (1.2 µg/L) treated groups, the liver architecture was found to be almost similar to that of control as shown in Fig. 5(c and d), as indicated by the regular arrangement of hepatocytes with well-defined sinusoids, except mild lymphatic infiltration in the sinusoids and the relatively lower DTC value (11.22 ± 0.66) as revealed in Fig. 3, Table 1 depicting slight damage to the organ. Furthermore, the fish liver exposed to 2 µg/L of FEN revealed irreparable damage to the liver as described by the DTC value 117.12 ± 1.27 presented in Fig. 3, Table 1, which is 62.29-fold higher than that of control (1.88 ± 0.26). The observed toxicopathic lesions in Fig. 5(e and f) are represented by perivascular oedema and lymphatic cuffing, necrosis, hydropic swelling, heavy lymphatic infiltration in the blood vessels, nuclear polymorphism and pycnotic nuclei. Nevertheless, concomitant treatment of AA with FEN (2 µg/L), showed marked improvement in the hepatic histological alterations as compared to that of the FEN alone treated group. However, vacuolisation was still registered at some places in the liver tissue (Fig. 5(g and h). The DTC value (16.16 ± 0.66) in Fig. 3, Table 1 showed a 3-fold decrease in the damage as compared to the pesticide-alone-treated group.

### 3.4 Ultrastructural analysis

**Fig. 6:**
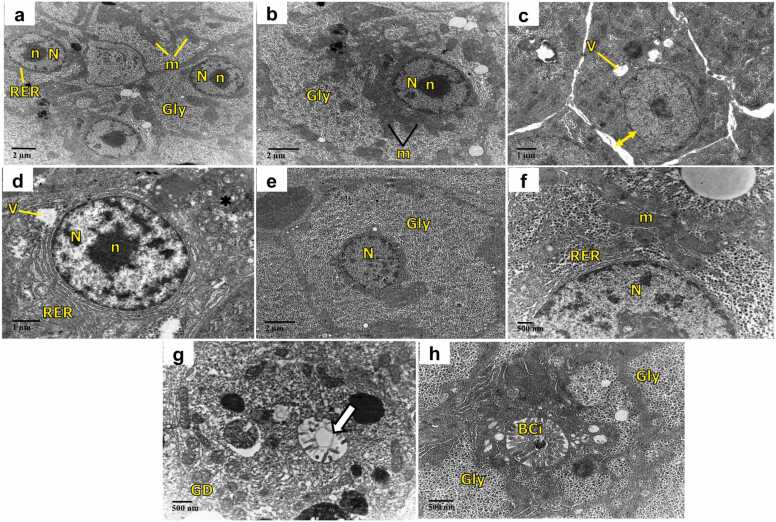
Transmission electron micrographs of liver of *C. idella*: (a) control and (b) AA alone treated group showing normal hepatocytes including a large centrally placed nucleus with nucleolus surrounded by rough endoplasmic reticulum and mitochondria along with abundant glycogen granules in the cytoplasm; (c-h) exposed to FEN and orally administered with AA for 15 days: (c, d) 1.2 µg/L FEN exposure showing dilated intercellular spaces (line arrow: double), vacuolization and dilation of rough endoplasmic reticulum; (e, f) treatment with AA + 1.2 µg/L FEN. Note no vacuolization in the hepatocytes. (g) 2 µg/L FEN exposure showing glycogen depletion, damaged bile canaliculi (white block arrow); (h) treatment with AA + 2 µg/L FEN showing the normal structure of bile canaliculi and abundant glycogen granules; nucleus (N), nucleolus (n), glycogen (Gly), rough endoplasmic reticulum (RER), mitochondria (m), vacuolization (V), glycogen depletion (GD), bile canaliculi (BCi).

TEM studies performed on the liver of control and AA alone treated fish as appeared in Fig. 6(a and b) manifested hepatocytes with centrally located rounded nucleus with abundant euchromatin, few condensed heterochromatin at the periphery, and a central prominent nucleolus. The nucleus is surrounded by parallelly arranged rough endoplasmic reticulum well arranged in parallel layers and mitochondria with dense matrices. A variable amount of glycogen randomly located throughout the cytoplasm was also evident.

In Fig. 6(c and d) after 15 days of exposure to FEN (1.2 µg/L), hepatic cells displayed increased intercellular spaces, vacuolization and dilation of RER. In addition to these changes in Fig. 6(g), depletion of glycogen and distorted microvilli of bile canaliculi were observed in the liver of fish exposed to FEN (2 µg/L). On the other hand, treatment of AA along with both the concentrations of FEN (Fig. 6e, f, and h) retained the normal structure of hepatocytes with abundant glycogen granules scattered throughout the cytoplasm with the normal structure of bile canaliculi.

**Fig. 7:**
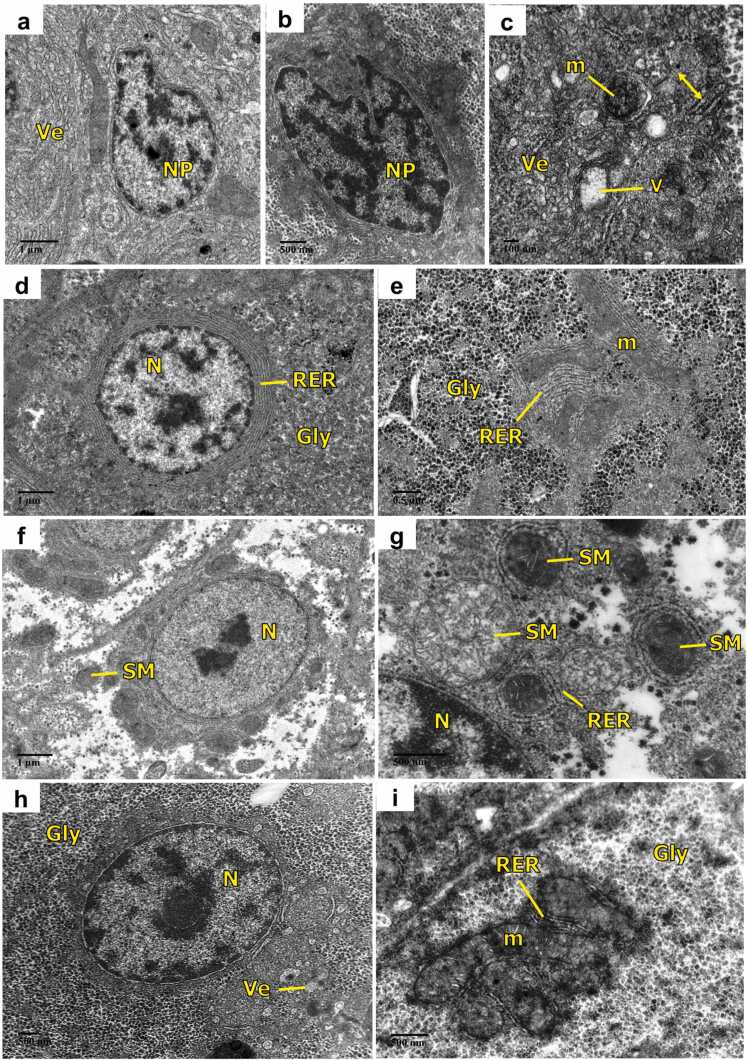
Transmission electron micrographs of liver of *C. idella* exposed to FEN and orally administered with AA (30 days): (a-c) 1.2 µg/L FEN exposure showing different types of abnormal nuclei demonstrating nuclear polymorphism, increased vesiculation, damaged mitochondria, dilation in the endoplasmic reticulum (line arrow: double) and vacuolization; (d, e) treatment with AA + 1.2 µg/L FEN. Note normal structure of nucleus surrounded by rough endoplasmic reticulum and mitochondria. (f, g) 2 µg/L FEN exposure showing necrotic hepatocytes with cytoplasmic disruption resulting in loss of cellular organelles except for endoplasmic reticulum, swollen mitochondria with severe dilation in the cristae; (h, i) treatment with AA + 2 µg/L FEN showing normal hepatocytes with abundant glycogen granules and mitochondria with clear cristae, except some vesiculation in the endoplasmic reticulum; nuclear polymorphism (NP), vesiculation (Ve), mitochondria (m), vacuolization (V), nucleus (N), glycogen (Gly), rough endoplasmic reticulum (RER), swollen mitochondria (SM).

After 30 days, lower concentration of the pesticide as shown in Fig. 7(a-c), displayed different types of abnormal nuclei in hepatocytes demonstrating nuclear polymorphism, increased dilation and vesiculation of endoplasmic reticulum, damaged mitochondria and vacuolization. However, exposure to the higher concentration of FEN, Fig. 7(f and g) presented necrotic hepatocytes with cytoplasmic disruption resulting in loss of cellular organelles except for mitochondria and endoplasmic reticulum. Swollen mitochondria with severe dilation in the cristae were also seen. Further, supplementation of AA in both the FEN-exposed groups recovered the ultrastructure of hepatocytes as manifested by the presence of abundant glycogen granules and typical nucleus surrounded by RER and mitochondria with clear cristae (Fig. 7(d, e, h, and i). The small number of vesicles in Fig. 7(h) was also evident in the endoplasmic reticulum.

**Fig. 8:**
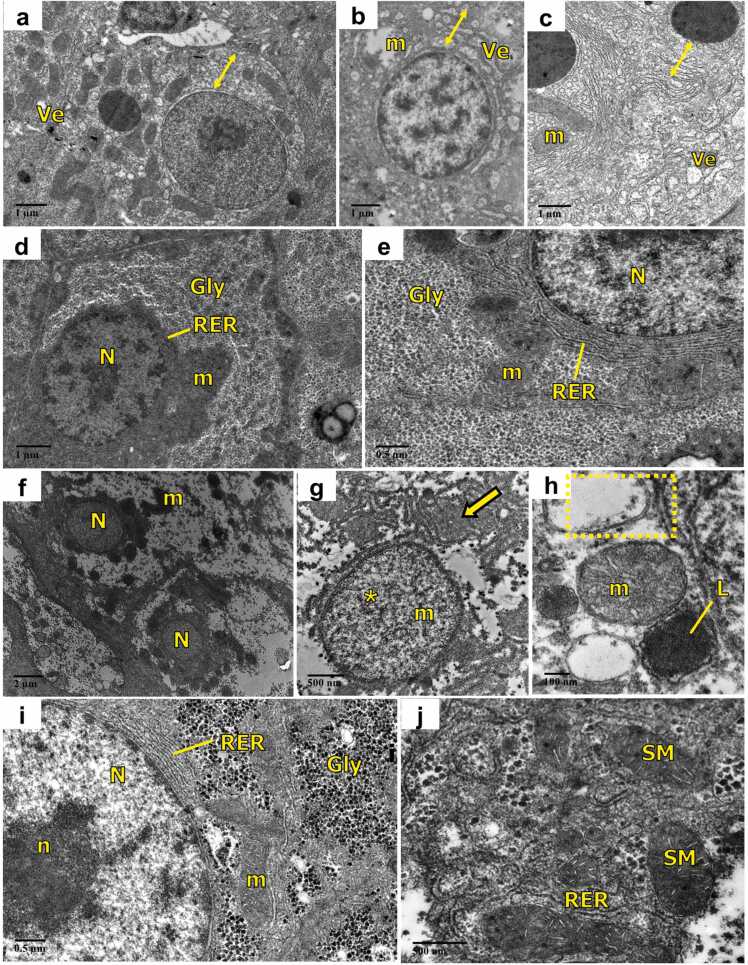
Transmission electron micrographs of liver of *C. idella* exposed to FEN and orally administered with AA (60 days): (a-c) 1.2 µg/L FEN exposure showing dilation of nuclear envelop (line arrow: double), dilation and vesiculation of endoplasmic reticulum, distorted mitochondria; (d, e) treatment with AA + 1.2 µg/L FEN showing hepatocytes with normal nucleus and conspicuous cytoplasm with normal mitochondria and RER; (f-h) 2 µg/L FEN showing severe necrosis with complete loss of cytoplasm and cell organelle, only remnants of mitochondria are present, complete loss of heterochromatin (*) dissolving cristae of mitochondria, whirling of endoplasmic reticulum (dotted rectangle); presence of lysosomes; (i, j) treatment with AA + 2 µg/L FEN showing hepatocyte with glycogen granules, normal nucleus, RER and mitochondria with clear cristae except some swollen mitochondria; vesiculation (Ve), mitochondria (m), nucleus (N), nucleolus (n), glycogen (Gly), rough endoplasmic reticulum (RER), lysosomes (L), swollen mitochondria (SM).

Long-term exposure (60 days) to FEN promotes degenerative changes in hepatocytes. As displayed in Fig. 8(a-c),exposure to 1.2 µg/L FEN exhibited dilation of nuclear envelope around the nucleus, severe dilation and vesiculation of endoplasmic reticulum, distorted mitochondria. However, treatment with AA and FEN demonstrated marked recovery in the ultrastructure of hepatocytes. The hepatocytes in Fig. 8(d and e) presented organised organelles and abundant glycogen in the cytoplasm, centrally located nucleus with conspicuous nucleolus and normal mitochondria with clear cristae. Exposure to 2 µg/L FEN as displayed in Fig. 8(f-h) resulted in severe necrosis of hepatocytes with complete loss of cytoplasm and cell organelle, only remnants of mitochondria are present. Complete loss of heterochromatin in the nucleus was also observed. The hepatocytes deficient of endoplasmic reticulum cisternae instead depicted fragmentation and whirling of rough endoplasmic reticulum. Hydropic mitochondria with dissolving cristae and lysosomes were evident. While, fish treated with AA and FEN, revealed marked improvement in the structure of hepatocytes with normal nucleus, RER, dense cytoplasm bearing glycogen granules and mitochondria with clear cristae as shown in Fig. 8(i and j). Some mitochondria were swollen, but still, possess organised cristae (Fig. 8j).

## 4 Discussion

Fenvalerate, a widely used synthetic pyrethroid, has been extensively studied for its impact on various biological systems. Our study delves into the intricate interactions between fenvalerate exposure and the biochemical, antioxidative, and histopathological aspects of liver function in *C. idella*. However, the incorporation of ascorbic acid, a well-known antioxidant, into the study framework introduces a novel dimension aimed at elucidating protective mechanisms against fenvalerate-induced hepatic toxicity. Liver is the vital organ for xenobiotics detoxification and is extensively used as a valuable biomarker for analysing the pathologies inflicted by various environmental pollutants in both field and lab analyses [44]. The hepatic ability to metabolise and reduce the toxicity of various xenobiotics is outweighed by the increased concentrations of these compounds, ultimately leading to pathological tissue changes [45]. In this study, FEN exposure significantly elevated serum levels of GOT, GPT, and ALP, indicating hepatic damage and increased protein catabolism. Elevated transaminase activities are consistent with liver cell injury, as these enzymes leak into the bloodstream due to hepatocellular damage and changes in membrane permeability. This elevation is a common response to various toxicants, as seen in previous studies on different fish species [46], [47].

Reactive oxygen species (ROS) can be generated from several sources, including mitochondrial electron transport, enzymatic reactions, and redox cycling, and their levels can be further amplified by xenobiotics [48]. FEN exposure in fish elevates ROS production, leading to oxidative stress and damage [49]. Fish play a crucial role as indicators of environmental stress, rely on antioxidant defenses (such as CAT, SOD, GPx, GST, and GSH enzymes) to neutralize ROS toxicity [50]. Among these, SOD and CAT play primary roles, with SOD converting superoxides produced in mitochondrial electron transport chains into hydrogen peroxide, further metabolized by microsomal SOD [51]. Catalase, a peroxisomal enzyme, mitigates oxidative stress by catalyzing the decomposition of hydrogen peroxide into water and molecular oxygen. Additionally, it can function as a peroxidase, utilizing hydrogen peroxide as an electron acceptor in oxidative reactions [52]. Additionally, the glutathione system comprising GPx, GST, and GSH is essential for reducing hydrogen peroxide to water and converting lipid hydroperoxides to alcohols, thereby protecting cellular integrity [53].

The study also highlighted the disruption of antioxidant defense mechanisms in FEN-exposed fish. Initial FEN exposure increased the activities of SOD, CAT, GSH, GST, and GPx, suggesting an adaptive response to counteract ROS overproduction. However, prolonged exposure led to a decrease in these antioxidant enzyme activities, indicating enzyme inhibition or failure to manage the oxidative stress. This resulted in elevated MDA levels, a marker of lipid peroxidation and oxidative liver damage. Similar findings were reported by Korkmaz et al. [45], who studied the effects of permethrin on the fish, *Cyprinus carpio*. They suggested that the pathogenicity observed was due to the formation of free radicals from permethrin metabolism, which involves hydrolytic ester cleavage and oxidation by cytochrome P450 microsomal enzymes in the liver. This process likely leads to a decrease in P450 enzyme levels, resulting in oxidative damage that manifests as a reduction in CAT activity and an increase in MDA levels, ultimately causing liver deterioration and necrosis [54]. Furthermore, Yang et al. [55] reported that the detoxification capacity of the glutathione system in tissues relies on the activities of peroxidase, glutathione reductase, and enzymes of the pentose phosphate pathway. In some situations, the activity of CAT is inhibited, while glutathione reductase and GPx remain unaffected. In such a situation, the glutathione system cannot cope with the extra load caused by the inhibition of CAT. Consequently, there is increased utilization of GSH, thus resulting in its reduction. Alternately, the depletion in GSH level might be explained by its participation in ROS removal, performing as both an oxygen radical scavenger and a substrate for various enzymes.

Histopathological and ultrastructural analyses revealed severe liver damage in FEN-exposed fish, including hydropic degeneration, necrosis, vacuolization, and lymphocytic infiltration. These lesions are indicative of hepatotoxicity and are align with studies on other fish species exposed to different insecticides [19], [56]. The observed liver pathologies suggest that FEN exposure disrupts normal hepatic function, leading to significant tissue damage which will be supported by the elevated serum levels of ALP, GPT, and GPT in the present study. Corroborating our results, fragmentation of hepatic mass and focal necrosis has also been reported by Ghayyur et al. [57] in *Cirrhinus mrigala* exposed to dimethoate, chlorpyrifos, and acetamiprid; Hasan et al. [58] in thiamethoxam-exposed *Trigogaster fasciata*.

In the present investigation, upon short-term FEN exposure, karyorrhexis and karyolysis were frequently observed which may be considered to be the initial steps towards necrosis or apoptosis [59]. Whereas upon prolonged exposure to the pesticide, more conspicuous alterations such as hepatic necrosis, perivascular oedema and lymphatic cuffing, hydropic swelling, heavy lymphatic infiltration in the blood vessels, nuclear polymorphism and pycnotic nuclei were observed. Necrosis of hepatocytes, a common pathological response, has also been observed in fish exposed to various pesticides such as methyl parathion and chlorpyrifos by Fanta et al. [60] and Verma et al. [61] respectively. Necrosis is considered to represent an array of disorders including altered enzyme activities, protein synthetic machinery, carbohydrate metabolism and loss of cell membrane integrity [62]. Necrosis has been associated with oxidative stress [16], which is also supported by the enhanced MDA level observed in the liver of the fish investigated in the present study.

Amongst various liver pathologies, vacuolization of the hepatocytes was encountered more frequently, and their occurrence increased with the increase in exposure to the pesticide. This vacuolization is a signal of lipid accumulation which may be due to the impaired synthesis of substances that aid in the transportation of hepatic triglycerides to the systemic circulation, thus indicating metabolic damage to the tissue [63]. According to Shah and Parveen [64] hepatic vacuolization may also indicate a defense mechanism to sequester the lipid-soluble contaminants, thereby reducing their toxic effects. Randomly distributed lipid vacuoles are also believed to be a pre-necrotic stage and are commonly observed in the hepatocyte cytoplasm of fish exposed to pesticide, chlorpyrifos [65].

Ascorbic acid (AA), a well-known water-soluble antioxidant, plays a crucial role in maintenance of fish physiology [28]. The hepatoprotective mechanism of AA likely involves several pathways. Firstly, AA enhances tissue thiol pools, which reduces the oxidative modification of antioxidant enzymes. Secondly, it acts as a chelating agent, binding to reactive byproducts of LPO and preventing further damage [66]. This study demonstrated that AA supplementation not only normalized the activities of serum markers like GOT, GPT, and ALP but also restored antioxidant enzyme function while significantly reducing histopathological alterations in fish exposed to FEN. The results are consistent with findings from Hathout et al. [11], who observed similar improvements in antioxidant activity in *Oreochromis niloticus* exposed to acetamiprid, when supplemented with AA.

Mirvaghefi et al. [67] reported that AA normalized SOD and CAT activity in *Oncorhynchus mykiss* exposed to diazinon, underscoring its role in mitigating oxidative stress by balancing the biochemical reactions associated with ROS production. The antioxidants electronegative properties allow it to act as an inhibitor, reviver, and scavenger of free radicals [68], reducing the overall rate of ROS production and enhancing cellular resilience. The present findings further align with previous work by Grajeda-Cota et al. [69] and Nakagawa et al. [70], who reported that AA supplementation decreased GSH utilization in hepatic cells by mitigating membrane oxidation and synergizing with other antioxidants like vitamin E and GSH. El-Garawani et al. [29] demonstrated the significant ameliorative effect of ascorbic acid on the liver of *Oreochromis niloticus* exposed to sub-lethal concentrations of imidacloprid. Insecticide exposure resulted in elevated oxidative stress, as evidenced by the increased activities of key antioxidant enzymes, including SOD, CAT, and GPX, along with higher lipid peroxidation levels. These changes indicate an imbalance in the antioxidant defense system, suggesting that insecticide triggers oxidative damage in fish liver. However, co-administration of AA with imidacloprid led to a significant restoration of the antioxidant profile. The activities of SOD, CAT, and GPX were effectively normalized, indicating that AA plays a vital role in reducing oxidative stress by neutralizing ROS generated during imidacloprid exposure. The recovery of these enzymatic activities underscores the protective efficacy of AA in maintaining liver function and mitigating the oxidative modifications induced by insecticide.

One of the more intriguing findings of our study is the reduction in autophagic vacuole formation within hepatocytes, as observed through electron microscopy. FEN intoxication typically disrupts autophagic processes, which are vital for cellular homeostasis. However, AA supplementation appeared to normalize these processes. Kurahashi et al. [71] similarly reported that AA restored the autophagic and cysteine transport systems in animals treated with acetaminophen, linking AA’s antioxidant properties to improved GSH production. This suggests that AA not only scavenges ROS but also indirectly supports critical detoxification pathways.

## 5 Conclusion

In conclusion, ascorbic acid (AA) has demonstrated substantial hepatoprotective effects in *Ctenopharyngodon idella* exposed to fenvalerate. AA treatment effectively normalized antioxidant enzyme activities, reduced oxidative stress markers, and mitigated histopathological damage in the liver, underscoring its potential as a therapeutic agent against pyrethroid-induced hepatotoxicity. Moreover, serum biochemical parameters, including SGOT, SGPT, and ALP levels, were positively modulated, indicating improved hepatic function and metabolic stability with AA supplementation. These results align with a significant body of research supporting AA’s role in counteracting xenobiotic-induced oxidative damage in diverse species and toxicants. The enhancement of tissue thiol reserves and decreased oxidative modification of enzymes appear to be core components of AA's protective mechanisms, suggesting its valuable application as a dietary supplement in aquaculture.

## Ethics statement

All experimental procedures involving fish were conducted following ethical guidelines and approved by Institutional Animal Ethics Committee, Panjab University, Chandigarh, under approval number PU/IAEC/S/14/159.

## Funding

Financial support from University Grant Commission, India (UGC-BSR -Grant No. F.7- 150/2007(BSR) dated 1-02-2013) in the form of Senior Research Fellowship to one of the authors, Smriti Batoye is duly acknowledged.

## CRediT authorship contribution statement

**Batoye Smriti:** Writing – original draft, Visualization, Validation, Methodology, Investigation, Funding acquisition, Formal analysis, Data curation, Conceptualization. **Verma Sakshi:** Writing – review & editing, Methodology. **Jindal Rajinder:** Writing – review & editing, Validation, Supervision, Resources. **Srivastava Nidhi:** Writing – review & editing.

## Declaration of Competing Interest

The authors declare that they have no known competing financial interests or personal relationships that could have appeared to influence the work reported in this paper.

## Data Availability

No data was used for the research described in the article.

## References

[1] Yao C., Huang L., Li C., Nie D., Chen Y., Guo X., Pang S. (2022). Exposure to fenvalerate and tebuconazole exhibits combined acute toxicity in zebrafish and behavioral abnormalities in larvae. Front. Environ. Sci..

[2] Farag M.R., Alagawany M., Bilal R.M., Gewida A.G., Dhama K., Abdel-Latif H.M., Naiel M.A. (2021). An overview on the potential hazards of pyrethroid insecticides in fish, with special emphasis on cypermethrin toxicity. Animals.

[3] Venturini F.P., de Moraes F.D., Rossi P.A., Avilez I.M., Shiogiri N.S., Moraes G. (2019). A multi-biomarker approach to lambda-cyhalothrin effects on the freshwater teleost matrinxa Brycon amazonicus: single-pulse exposure and recovery. Fish. Physiol. Biochem..

[4] Muggelberg L.L., Hartz K.E.H., Nutile S.A., Harwood A.D., Heim J.R., Derby A.P., Lydy M.J. (2017). Do pyrethroid-resistant Hyalella azteca have greater bioaccumulation potential compared to non-resistant populations? Implications for bioaccumulation in fish. Environ. Pollut..

[5] Aznar-Alemany Ò., Giménez J., de Stephanis R., Eljarrat E., Barceló D. (2017). Insecticide pyrethroids in liver of striped dolphin from the Mediterranean Sea. Environ. Pollut..

[6] Fırat Ö., Cogun H.Y., Yüzereroğlu T.A., Gök G., Fırat Ö., Kargin F., Kötemen Y. (2011). A comparative study on the effects of a pesticide (cypermethrin) and two metals (copper, lead) to serum biochemistry of Nile tilapia, Oreochromis niloticus. Fish. Physiol. Biochem..

[7] Xu R., Zheng R., Wang Y., Ma R., Tong G., Wei X., Hu K. (2021). Transcriptome analysis to elucidate the toxicity mechanisms of fenvalerate, sulfide gatifloxacin, and ridomil on the hepatopancreas of Procambarus clarkii. Fish. Shellfish Immunol..

[8] de Moraes F.D., Venturini F.P., Rossi P.A., Avilez I.M., da Silva de Souza N.E., Moraes G. (2018). Assessment of biomarkers in the neotropical fish Brycon amazonicus exposed to cypermethrin-based insecticide. Ecotoxicology.

[9] Hussein H.K., Elnaggar M.H., Al-Dailamy J.M. (2012). Protective role of Vitamin C against hepatorenal toxicity of fenvalerate in male rats. Glob. Adv. Res. J. Environ. Sci. Toxicol..

[10] Sandoval-Herrera N., Mena F., Espinoza M., Romero A. (2019). Neurotoxicity of organophosphate pesticides could reduce the ability of fish to escape predation under low doses of exposure. Sci. Rep..

[11] Hathout H.M., Sobhy H.M., Abou-Ghanima S., El-Garawani I.M. (2021). Ameliorative role of ascorbic acid on the oxidative stress and genotoxicity induced by acetamiprid in Nile tilapia (Oreochromis niloticus). Environ. Sci. Pollut. Res..

[12] Nataraj B., Hemalatha D., Rangasamy B., Maharajan K., Ramesh M. (2017). Hepatic oxidative stress, genotoxicity and histopathological alteration in fresh water fish Labeo rohita exposed to organophosphorus pesticide profenofos. Biocatal. Agric. Biotechnol..

[13] Ensibi C., Pérez-López M., Rodríguez F.S., Míguez-Santiyán M.P., Yahya M.D., Hernández-Moreno D. (2013). ). Effects of deltamethrin on biometric parameters and liver biomarkers in common carp (Cyprinus carpio L.). Environ. Toxicol. Pharmacol..

[14] van der Oost R., Beyer J., Vermeulen N.P.E. (2003). Fish bioaccumulation and biomarkers in environmental risk assessment: a review. Environ. Toxicol. Pharmacol..

[15] Mierzejewski J., Haney D.C., van den Hurk P. (2014). Biomarker responses in sunfish species and largemouth bass from the Saluda River, South Carolina. Ecotoxicol. Environ. Saf..

[16] Loganathan K., Tennyson S., Arivoli S. (2024). Triazophos toxicity induced histological abnormalities in Heteropneustes fossilis Bloch 1794 (Siluriformes: Heteropneustidae) organs and assessment of recovery response. J. Basic Appl. Zool..

[17] Seth N., Saxena K.K. (2003). Hematological responses in a freshwater fish Channa punctatus due to fenvalerate. Bull. Environ. Contam. Toxicol..

[18] Tripathi G., Verma P. (2004). Fenvalerate-induced changes in a catfish, Clarias batrachus: metabolic enzymes, RNA and protein. Comp. Biochem. Physiol. Part C: Toxicol. Pharmacol..

[19] Kaval Oğuz E., Alkan Z., Oğuz A.R., Ergöz Azizoğlu B., Örgi E. (2024). Histopathological determination of changes in tissues of Lake Van Fish (Alburnus tarichi (Güldenstädt, 1814)) exposed to Esfenvalerate. Chem. Ecol..

[20] An X., Di S., Wang X., Cao C., Wang D., Chen L., Wang Y. (2024). Combined toxicity of aflatoxin B1 and tebuconazole to the embryo development of zebrafish (Danio rerio). Chemosphere.

[21] Mushigeri S.B., David M. (2005). Fenvalerate induced changes in the Ach and associated AchE activity in different tissues of fish Cirrhinus mrigala (Hamilton) under lethal and sub-lethal exposure period. Environ. Toxicol. Pharmacol..

[22] Prusty A.K., Kohli M.P.S., Sahu N.P., Pal A.K., Saharan N., Mohapatra S., Gupta S.K. (2011). Effect of short term exposure of fenvalerate on biochemical and haematological responses in Labeo rohita (Hamilton) fingerlings. Pestic. Biochem. Physiol..

[23] Zhang L., Hong X., Yan S., Zha J. (2022). Environmentally relevant concentrations of fenvalerate induces immunotoxicity and reduces pathogen resistance in Chinese rare minnow (Gobiocypris rarus). Sci. Total Environ..

[24] Fetoui H., Makni M., Garoui E.M., Zeghal N. (2010). Toxic effects of lambda-cyhalothrin, a synthetic pyrethroid pesticide, on the rat kidney: involvement of oxidative stress and protective role of ascorbic acid. Exp. Toxicol. Pathol..

[25] Gęgotek A., Skrzydlewska E. (2022). Antioxidative and anti-inflammatory activity of ascorbic acid. Antioxidants.

[26] Zeng X., Du Z., Ding X., Jiang W. (2021). Protective effects of dietary flavonoids against pesticide-induced toxicity: a review. Trends Food Sci. Technol..

[27] Garcia F., Pilarski F., Onaka E.M., de Moraes F.R., Martins M.L. (2007). Hematology of Piaractus mesopotamicus fed diets supplemented with vitamins C and E, challenged by Aeromonas hydrophila. Aquaculture.

[28] Rathore S.S., Hanumappa S.M., Yusufzai S.I., Suyani N.K., Abdullah-Al-Mamun M., Nasren S., Kalyani R. (2023). Dietary administration of engineered nano-selenium and vitamin C ameliorates immune response, nutritional physiology, oxidative stress, and resistance against Aeromonas hydrophila in Nile Tilapia (Oreochromis niloticus). Biol. Trace Elem. Res..

[29] El-Garawani, I.M., Khallaf, E.A., Elgendy, R.G., Mersal, G.A., & El-Seedi, H.R.. (2021). Imidacloprid Induces Oxidative Stress and Genotoxicity in Nile Tilapia: The Role of Ascorbic Acid Combined Exposure. 10.1038/s41598-021-94020-y PMC8289846 34282219

[30] Paduraru E., Flocea E.I., Lazado C.C., Simionov I.A., Nicoara M., Ciobica A., Jijie R. (2021). Vitamin C mitigates oxidative stress and behavioral impairments induced by deltamethrin and lead toxicity in zebrafish. Int. J. Mol. Sci..

[31] Robea M.A., Jijie R., Nicoara M., Plavan G., Ciobica A.S., Solcan C., Strungaru S.A. (2020). Vitamin C attenuates oxidative stress and behavioral abnormalities triggered by fipronil and pyriproxyfen insecticide chronic exposure on zebrafish juvenile. Antioxidants.

[32] Batoye S., Jindal R., Verma S. (2021). Ameliorating effect of ascorbic acid on fenvalerate induced ultrastructural changes in scales, erythrocytes and gills of Ctenopharyngodon idella (Valenciennes, 1844). Environ. Sci. Pollut. Res..

[33] Kono Y. (1978). Generation of superoxide radical during autoxidation of hydroxylamine and an assay for superoxide dismutase. Arch. Biochem. Biophys..

[34] Luck H., Bergmeyer H.O. (1971).

[35] Mohandas J., Marshall J.J., Duggin G.G., Horvath J.S., Tiller D.J. (1984). Low activities of glutathione-related enzymes as factors in the genesis of urinary bladder cancer. Cancer Res..

[36] Habig W.H., Pabst M.J., Jakoby W.B. (1974). Glutathione S-transferases: the first enzymatic step in mercapturic acid formation. J. Biol. Chem..

[37] Beutler E., Duron O., Kelly B.M. (1963). Improved method for the determination of blood glutathione. J. Lab. Clin. Med..

[38] Buege J.A., Aust S.D. (1978).

[39] Lowry O.H., Rosebrough N.J., Farr A.L., Randall R.J. (1951). Protein measurement with the Folin phenol reagent. J. Biol. Chem..

[40] Cengiz E.I., Unlu E. (2006). Sublethal effects of commercial deltamethrin on the structure of the gill, liver and gut tissues of mosquitofish, Gambusia affinis: a microscopic study. Environ. Toxicol. Pharmacol..

[41] Poleksic, V., & Mitrovic-Tutundzic, V.. (1994). Fish gills as a monitor of sublethal and chronic effects of pollution (pp. 339-352). on freshwater fish. Oxford, London, UK: Fishing News books.

[42] Peng J., Singh A., Ireland W.P., Chu I. (1997). Polychlorinated biphenyl congener 153-induced ultrastructural alterations in rat liver: a quantitative study. Toxicology.

[43] Camargo M.M., Martinez C.B. (2007). Histopathology of gills, kidney and liver of a Neotropical fish caged in an urban stream. Neotrop. Ichthyol..

[44] Mahmoud A.H., Darwish N.M., Kim Y.O., Viayaraghavan P., Kwon J.T., Na S.W., Kim H.J. (2020). Fenvalerate induced toxicity in Zebra fish, Danio rerio and analysis of biochemical changes and insights of digestive enzymes as important markers in risk assessment. J. King Saud. Univ. -Sci..

[45] Korkmaz N., Uğurer O., Örün İ. (2023). Toxic effects of the synthetic pyrethroid permethrin on the hematological parameters and antioxidant enzyme systems of the freshwater fish Cyprinus carpio L. Ecotoxicology.

[46] Yilmaz-Ozden T., Can A., Karatug A., Pala-Kara Z., Okyar A., Bolkent S. (2016). Carbon tetrachloride-induced kidney damage and protective effect of Amaranthus lividus L. in rats. Toxicol. Ind. Health.

[47] Hossain M.A., Sutradhar L., Sarker T.R., Saha S., Iqbal M.M. (2022). Toxic effects of chlorpyrifos on the growth, hematology, and different organs histopathology of Nile tilapia, Oreochromis niloticus. Saudi J. Biol. Sci..

[48] Narra M.R. (2017). Haematological and immune upshots in Clarias batrachus exposed to dimethoate and defying response of dietary ascorbic acid. Chemosphere.

[49] Wu S., Hu G., Zhao X., Wang Q., Jiang J. (2018). Synergistic potential of fenvalerate and triadimefon on endocrine disruption and oxidative stress during rare minnow embryo development. Environ. Toxicol..

[50] Veedu S.K., Ayyasamy G., Tamilselvan H., Ramesh M. (2022). Single and joint toxicity assessment of acetamiprid and thiamethoxam neonicotinoids pesticides on biochemical indices and antioxidant enzyme activities of a freshwater fish Catla catla. Comp. Biochem. Physiol. Part C: Toxicol. Pharmacol..

[51] Al-Ghanim K.A., Mahboob S., Vijayaraghavan P., Al-Misned F.A., Kim Y.O., Kim H.J. (2020). Sub-lethal effect of synthetic pyrethroid pesticide on metabolic enzymes and protein profile of non-target Zebra fish, Danio rerio. Saudi J. Biol. Sci..

[52] Clasen B., Loro V.L., Murussi C.R., Tiecher T.L., Moraes B., Zanella R. (2018). Bioaccumulation and oxidative stress caused by pesticides in Cyprinus carpio reared in a rice-fish system. Sci. Total Environ..

[53] Kong Y., Li M., Shan X., Wang G., Han G. (2021). Effects of deltamethrin subacute exposure in snakehead fish, Channa argus: biochemicals, antioxidants and immune responses. Ecotoxicol. Environ. Saf..

[54] Manna S., Bhattacharyya D., Mandal T.K., Das S. (2004). Repeated dose toxicity of alfa-cypermethrin in rats. J. Vet. Sci..

[55] Yang C., Lim W., Song G. (2020). Mediation of oxidative stress toxicity induced by pyrethroid pesticides in fish. Comp. Biochem. Physiol. Part C: Toxicol. Pharmacol..

[56] Farsani H.G., Rashidian G., Narimanizad F., Khodadadi M., Gerami M.H. (2015). Histopathology and biochemical analysis of common carp (Cyprinus carpio) exposed to sublethal concentrations of carboxin-thiram (vitavax thiram). J. Fish. Aquat. Sci..

[57] Ghayyur S., Khan M.F., Tabassum S., Ahmad M.S., Sajid M., Badshah K., Qamer S. (2021). A comparative study on the effects of selected pesticides on hemato-biochemistry and tissue histology of freshwater fish Cirrhinus mrigala (Hamilton, 1822). Saudi J. Biol. Sci..

[58] Hasan M.M., Uddin M.H., Islam M.J., Biswas S., Sumon K.A., Prodhan M.D.H., Rashid H. (2022). Histopathological alterations in liver and kidney tissues of banded gourami (Trichogaster fasciata) exposed to thiamethoxam. Aquac. Stud..

[59] Elmore S. (2007). Apoptosis: a review of programmed cell death. Toxicol. Pathol..

[60] Fanta E., Rios F.S.A., Romão S., Vianna A.C.C., Freiberger S. (2003). Histopathology of the fish Corydoras paleatus contaminated with sublethal levels of organophosphorus in water and food. Ecotoxicol. Environ. Saf..

[61] Verma D.K., Tripathi R., Dsa V.K., Pandey R.K. (2020). Histopathological changes in liver and kidney of Heteropneustes fossilis (Bloch) on chlorpyrifos exposure. Sci. Temper..

[62] Samuel T., Thresia M., Loganathan K., Arivoli S., Raveen R., William J. (2019). Histopathological responses of liver tissues of the African catfish Clarias gariepinus (Burchell, 1822)(Teleostei: Clariidae) to the biopesticide, azadirachtin. J. Environ. Bio-Sci..

[63] Mela M., Guiloski I.C., Doria H.B., Randi M.A.F., de Oliveira Ribeiro C.A., Pereira L., De Assis H.S. (2013). Effects of the herbicide atrazine in neotropical catfish (Rhamdia quelen). Ecotoxicol. Environ. Saf..

[64] Shah Z.U., Parveen S. (2022). Oxidative, biochemical and histopathological alterations in fishes from pesticide contaminated river Ganga, India. Sci. Rep..

[65] Al-Harbi M.S., El-Rahman F.A.A., El-Shenawy N.S., Al-Mutrafi W.M. (2014). The beneficial effects of ascorbic acid during chlorpyrifos-induced oxidative stress and histopathological changes in Oreochromis spilurus. Toxicol. Environ. Health Sci..

[66] Labh S.N. (2024). Vitamin C (L-ascorbate 2-triphosphate Calcium) enhances the growth, immunobiochemical, and haemato-morphological performance of common carp Cyprinus carpio. Adv. Obes. Weight Manag Control.

[67] Mirvaghefi A., Ali M., Poorbagher H. (2016). Effects of vitamin C on oxidative stress parameters in rainbow trout exposed to diazinon. Ege J. Fish. Aquat. Sci..

[68] Elzoghby R.R., Ahlam F.H., Abdel-Fatah A., Farouk M. (2014). Protective role of vitamin C and green tea extract on malathion-induced hepatotoxicity and nephrotoxicity in rats. Am. J. Pharmacol. Toxicol..

[69] Grajeda-Cota P., Ramírez-Mares M.V., de Mejía E.G. (2004). Vitamin C protects against in vitro cytotoxicity of cypermethrin in rat hepatocytes. Toxicol. Vitr..

[70] Nakagawa Y., Cotgreave I.A., Moldbus P. (1991). Relationships between ascorbic acid and a-tocopherol during diquat-induced redox cycling in isolated rat hepatocytes. Biochem. Pharmacol..

[71] Kurahashi T., Lee J., Nabeshima A., Homma T., Kang E.S., Saito Y., Fujii J. (2016). Ascorbic acid prevents acetaminophen-induced hepatotoxicity in mice by ameliorating glutathione recovery and autophagy. Arch. Biochem. Biophys..

